# Pelagic barite precipitation at micromolar ambient sulfate

**DOI:** 10.1038/s41467-017-01229-5

**Published:** 2017-11-07

**Authors:** Tristan J. Horner, Helena V. Pryer, Sune G. Nielsen, Peter W. Crockford, Julia M. Gauglitz, Boswell A. Wing, Richard D. Ricketts

**Affiliations:** 1 NIRVANA Laboratories, Woods Hole Oceanographic Institution, Woods Hole, MA 02543-1050 USA; 2 Department of Marine Chemistry and Geochemistry, Woods Hole Oceanographic Institution, Woods Hole, MA 02543-1050 USA; 3 Department of Geology and Geophysics, Woods Hole Oceanographic Institution, Woods Hole, MA 02543-1050 USA; 4 Department of Earth and Planetary Sciences, McGill University, Montreal, QC Canada H3A 0E8; 5 Geological Sciences, University of Colorado Boulder, Boulder, CO 80309 USA; 6 Large Lakes Observatory, University of Minnesota Duluth, Duluth, MN 55812 USA

## Abstract

Geochemical analyses of sedimentary barites (barium sulfates) in the geological record have yielded fundamental insights into the chemistry of the Archean environment and evolutionary origin of microbial metabolisms. However, the question of how barites were able to precipitate from a contemporary ocean that contained only trace amounts of sulfate remains controversial. Here we report dissolved and particulate multi-element and barium-isotopic data from Lake Superior that evidence pelagic barite precipitation at micromolar ambient sulfate. These pelagic barites likely precipitate within particle-associated microenvironments supplied with additional barium and sulfate ions derived from heterotrophic remineralization of organic matter. If active during the Archean, pelagic precipitation and subsequent sedimentation may account for the genesis of enigmatic barite deposits. Indeed, barium-isotopic analyses of barites from the Paleoarchean Dresser Formation are consistent with a pelagic mechanism of precipitation, which altogether offers a new paradigm for interpreting the temporal occurrence of barites in the geological record.

## Abstract (web summary)

The question of how significant barite deposits were able to form from early Earth’s low-sulfate seas remains controversial. Here, the authors show pelagic barite precipitation within a strongly barite-undersaturated ecosystem, highlighting the importance of particle-associated microenvironments.

## Introduction

The size and turnover rate of the marine sulfate reservoir is fundamentally linked to the global biogeochemical cycles of oxygen, nutrients, and carbon^1^. Since barite (barium sulfate, BaSO_4_) can record the input^2^, inventory^3^, and internal cycling^4^ of sulfate within the marine reservoir, the chemistry of sedimentary barites offer a powerful means to study the history of Earth’s biogeochemical cycles^5^. Furthermore, the extremely low solubility of barite at pressures and temperatures relevant to biogeochemical processes renders barite a robust archive of marine and atmospheric chemistry on timescales ranging from the Archean (4.0–2.5 Ga) to present^6,7^.

Broadly, three types of barite are recognized to contribute to sedimentary accumulations:^8^ hydrothermal^9^ or sedimentary^10^ exhalative, formed by injection of Ba-rich fluids into sulfate-bearing (sea) water, and vice versa; diagenetic, formed through substitution of Ba for calcium in evaporite sequences (often termed baritization; e.g., ref. ^9^); and pelagic (often termed marine), the dominant vector of particulate Ba in the modern oceans^11^. Pelagic barites precipitate authigenically in the upper water column as discrete μm-sized crystals that accumulate in seawater^12^ and on the seafloor^13^ beneath regions of high productivity^14^. Since modern seawater is slightly undersaturated with respect to barite^15^, pelagic barite precipitation is thought to occur in ephemeral, particle-associated microenvironments that achieve barite saturation during microbial oxidation of organic matter^16^.

Given these three precipitation pathways, it is often assumed that at times in Earth’s past when the oceans’ sulfate concentrations were orders of magnitude lower than today, such as during Archean Aeon (approximately micromolar sulfate^17–19^), pelagic barite precipitation constituted a negligible proportion of total barite sedimentation. However, the existence of Archean barite deposits that are not easily classified into recognized exhalative or diagenetic models (e.g., refs. ^6,20^) requires that either barite depositional pathways were fundamentally different in the past, or that pelagic precipitation—coupled to microbial respiration of organic matter—did in fact contribute to the sedimentation of enigmatic barites at micromolar ambient sulfate.

We tested the possibility of pelagic barite precipitation at micromolar ambient sulfate concentrations in a naturally sulfate-poor freshwater ecosystem, Lake Superior. The low-sulfate levels in this lake (40 μM^21^) contribute to an exceptionally barite-undersaturated water column, with *Ω*_barite_ = 0.02 (where ${{\Omega }}_{\rm{barite}} = ( {\{ {{\rm{Ba}}}^{2 + }} \} \cdot \{ {{\rm{SO}}_4^{2 - }} \} ){\rm{/}}K_{{\rm{sp}}}$). In contrast, modern seawater is generally close to saturation (*Ω*_barite_ = 1) and rarely <0.2^15^. Moreover, Lake Superior waters are thermally stratified, strongly oligotrophic^22^, phosphate-limited^23^, and possess a remarkably active cyanobacterial population^24^, with up to 50% of primary productivity attributable to autotrophic picoplankton^25^ (<3 μm). These features altogether render Lake Superior a powerful modern analog for studying Ba cycling in the trace-sulfate, barite-undersaturated conditions inferred for Earth’s earliest oceans.

Here, we present multi-element and barium-isotopic geochemical data evidencing pelagic barite precipitation in the barite-undersaturated water column of Lake Superior. These data require that pelagic precipitation take place within protected, particle-associated microenvironments that appear co-located with the strongest reductions in particulate organic matter concentrations. These patterns bear striking similarity to the open ocean and support a similar mechanism for low-sulfate environments whereby pelagic barite precipitation is closely coupled to heterotrophic remineralization of organic matter^16^. We augment these results with barium-isotopic analyses of the oldest barites on Earth—deposits from the Paleoarchaean Dresser Formation—that reveal compositions consistent with a pelagic origin. Overall, these discoveries attest to the biogeochemical significance of barite deposits and offer a novel framework for interpreting the genesis, temporal occurrence, and barium-isotopic composition of barites in the geological record.

## Results

### Multi-element and Ba-isotopic data

**Fig. 1:**
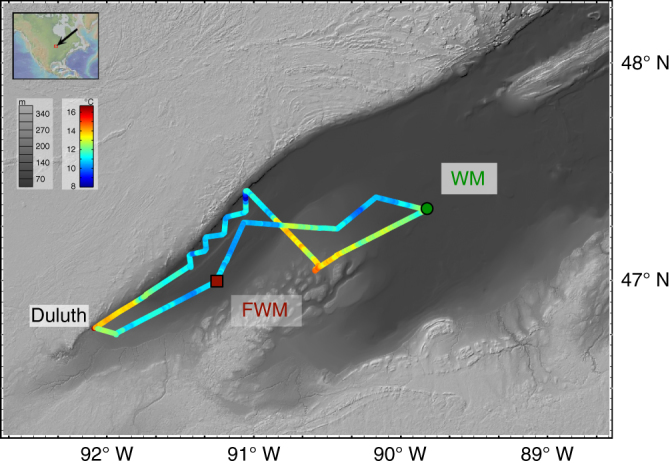
Map showing cruise track, underway temperature, and sampling locations in Lake Superior. Dissolved and particulate samples were collected at FWM (46°59′54.7″ N, 91°14′46.5″ W; 144 m water depth; 26 August 2015) and WM (47°19′53.8″ N, 89°49′17.0″ W; 180 m water depth; 27 August 2015) aboard the R/V *Blue Heron* (cruise BH15-11, Duluth MN–Duluth MN). Map drafted using GeoMapApp (http://www.geomapapp.org); Lake Superior bathymetry from National Centers for Environmental Information (https://www.ngdc.noaa.gov)

**Fig. 2:**
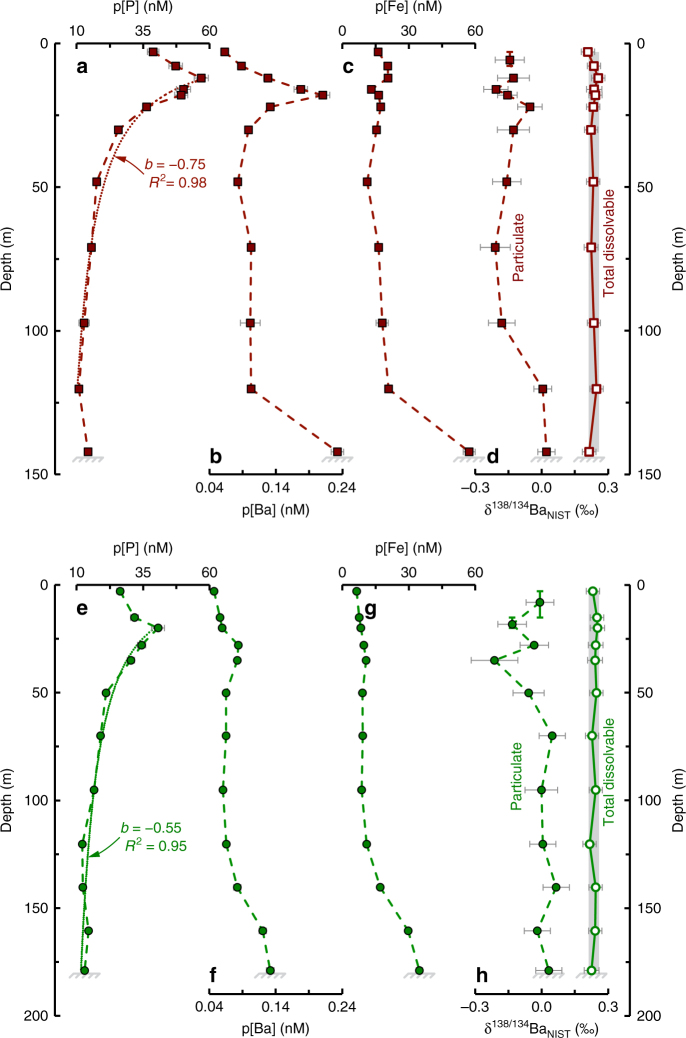
Multi-element geochemistry from Lake Superior. Particulate data were obtained by filtering between 0.5–3.0 L of Lake Superior water through a 0.45 μm polyethersulfone membrane filter immediately after collection. **a**–**d** from St. FWM; **e**–**h** from St. WM; dashed and solid lines show particulate and dissolved samples, respectively. Vertical error bars on the shallowest samples in **d**, **h** denote depth ranges over which particulate samples were pooled to obtain sufficient Ba for isotopic analysis. Horizontal error bars for any given property measurement reflect the propagated 2 × SD uncertainty. Thin dotted lines in **a**, **e** illustrate power-law fits to p[P] profiles^28^, excluding the benthic nepheloid layer sample from FWM (see text). Total dissolvable [Ba] and Ba-isotopic compositions are uniform for both stations at 69.7 ± 1.4 nM and +0.23 ± 0.02‰, respectively (±2 SD; *n* = 24); pBa constitutes 0.1–0.3% of total Ba

Two extensively studied stations in the western arm of Lake Superior were selected for geochemical characterization: FWM, a near-shore location and WM, an offshore station with lower surface productivity and terrigenous inputs compared to FWM (Fig. 1). Profiles of p[P] (particulate phosphorus), p[Ba], and p[Fe] (iron) all show depth-dependent patterns that bear remarkable similarity to the open ocean (see below; Fig. 2; e.g., ref. ^26^). Particulate [P], used here as a proxy for total particulate organic matter, is seen to increase with depth in the epilimnion, before sharply decreasing through the thermocline and hypolimnion. The depth-dependent pattern of organic matter attenuation seen in Lake Superior strongly resembles depth-normalized power-law functions used to fit profiles of organic matter reactivity in sediments underlying St. WM and FWM^27^ and of organic matter flux attenuation in the modern water column^28^. Using this framework and the maximum in p[P] as the reference depth, we calculate power-law exponents, *b*, of −0.75 (*R*^2^ = 0.98) for St. FWM and −0.55 for St. WM (*R*^2^ = 0.95). As with previous studies conducted during summer thermal stratification, maximum in p[P] is observed above the DCM (deep chlorophyll maximum; Supplementary Fig. 1, ref.^22^, suggesting that the DCM is not formed in situ but possibly maintained via loss of particulate organic matter through respiration^29^.

In addition to determining particulate multi-element geochemistry, we also determined stable Ba-isotopic compositions on dissolved and particulate samples from Lake Superior. Several recent studies demonstrated the sensitivity of Ba-isotopic compositions to barite cycling^30–33^ and thus—by analogy to other stable isotopic systems—insights into the environments hosting and pathways governing (barite) mineral precipitation:

1 $${{{ {\delta}}} ^{138/134}{\rm{Ba}}_{\rm{NIST}} = {\left(\left({\,}^{138}{\rm{Ba}/}^{134}{\rm{Ba}}\right)_{\rm{sample}}/\left( {\,}^{138}{\rm{Ba/}}^{134}{\rm{Ba}}\right)_{{\rm{NIST}}\,{\rm{SRM}}\,{\rm{3104a}}} - {1}\right)} \times 1000}$$

Particulate [Ba] and Ba-isotopic patterns are similar at FWM and WM: near-surface particulates are characterized by variably light Ba-isotopic compositions, whereas deeper particulates possess heavier δ^138/134^Ba_NIST_ and span a narrower range of values. These variations must reflect differences unique to the particulate phase as total dissolvable (i.e., unfiltered, which closely approximates dissolved) Ba concentrations and Ba-isotopic compositions are depth-invariant (Fig. 2d, h; see “Methods” section). For both FWM and WM, p[Ba] is lowest at the surface, increasing to an inflection point ~20–30 m below the surface, 6–8 m below peak p[P], then decreases with depth (Fig. 2b, f), akin to profiles from the open ocean^12,26^. A second p[Ba] maximum is observed in near-bottom samples from both stations, co-located with a deep turbidity feature and maximum in p[Fe](Fig. 2c, h; Supplementary Fig. 1). Since particulate Fe concentrations primarily reflect the underlying distribution of terrigenous material rather than association with organic matter or precipitation of authigenic Fe-oxide minerals^34^, increased p[Fe] at the bottom depths of FWM and WM likely derives from the significant benthic nepheloid layer that is common to large parts of the lake during summer thermal stratification^35^. Moreover, Ba-isotopic compositions of the deepest particulate samples from both stations possess mean Ba-isotopic compositions = +0.02 ± 0.02‰ (±2 SE, *n* = 8), approaching the range of Ba-isotopic composition of silicate rocks of +0.10 ± 0.05‰ (±2SE, *n* = 9;^36^). The significantly heavier Ba-isotopic compositions of both the crustally derived and nepheloid layer particles, compared to overlying particles (mean = −0.13 ± 0.13, *n* = 14; two-way, unpaired *t*-test *p*~0.0001), combined with the p[Fe] data support the notion that the near-bottom p[Ba] feature derives from resuspension of lithogenic matter rather than in situ production.

### Partitioning of pBa excess

**Fig. 3:**
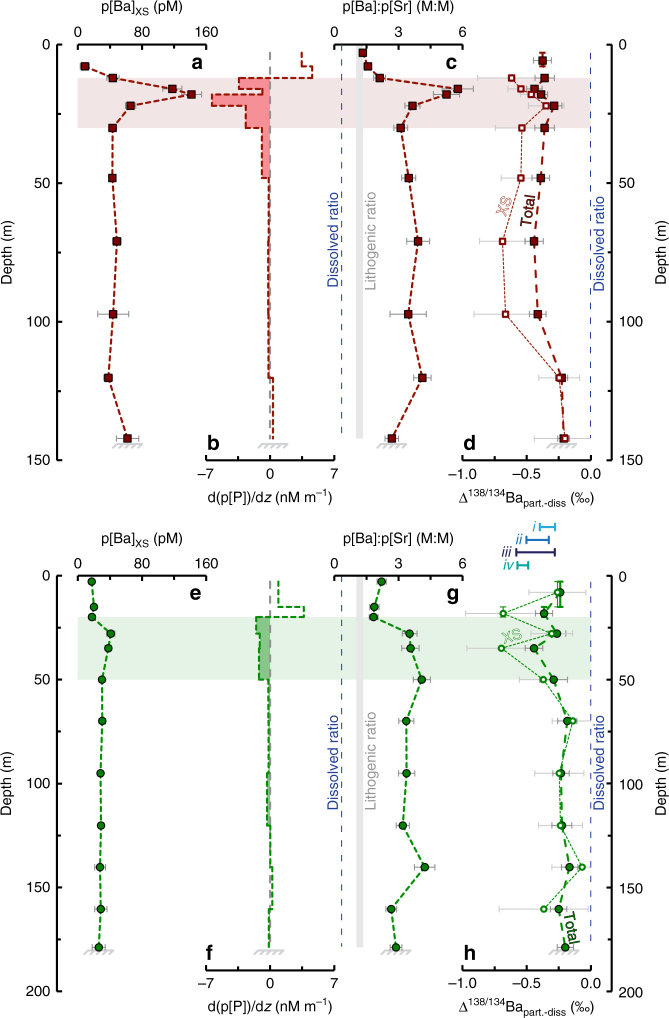
Characterization of the pBa excess in Lake Superior. **a**–**d** from St. FWM; **e**–**h** from St. WM. Depth profiles of **a**, **e** p[Ba]_XS_; **b**, **f** first derivative of p[P] with respect to depth, illustrating that the depths and magnitude of most intensive organic matter attenuation are correlated with the depths and magnitude of pBa_XS_; **c**, **g** particulate Ba:Sr ratios, also shown are the dissolved (dashed line) and average crustal ratio (vertical bar); **d**, **h** Ba-isotopic data expressed as the difference from dissolved Ba-isotopic compositions; Δ^138/134^Ba_part.−diss._ = δ^138/134^Ba_part._ − δ^138/134^Ba_diss._. Isotopic fractionation factors between particulate and dissolved Ba are shown as *i*, *ii*, *iii*, and *iv* for: inorganic barite precipitation^30^, mean Lake Superior p[Ba]_XS_ (this study), regression of seawater data^31–33^, and marine particles from the upper water column of the South Atlantic (Supplementary Fig. 7), respectively. Vertical error bars on the shallowest samples in **d**, **h** denote depth ranges over which particulate samples were pooled to obtain sufficient Ba for isotopic analysis. Horizontal error bars for any given property measurement reflect the propagated 2 × SD uncertainty. Shaded region highlights depth interval over which organic matter attenuation exceeds −1 nM P m^−1^

Unlike the deep p[Ba] maximum, we contend that the near-surface peak in p[Ba] is a consequence of in situ production. This near-surface peak in p[Ba] is not directly associated with organic matter (nor with other labile-type particulate elements; Supplementary Fig. 2); it is clearly discerned as a distinct feature below peak p[P] and similarly cannot be explained by the underlying distribution of particulate terrigenous material (Fig. 2; Supplementary Fig. 3). To quantitatively explore the nature of this near-surface p[Ba] feature, we partitioned our p[Ba] data into its constituent components, Ba that is: lithogenic-bound, associated with organic matter, and pBa_XS_ (i.e., excess; ref. ^37^), using:

2 $${\rm{p[Ba]}}_{{\rm{XS}}} = {\rm{p[Ba]}}_{{\rm{meas}}{\rm{.}}} - {\rm{p[Fe]}}_{{\rm{meas}}{\rm{.}}} \times \left( {\frac{{\rm{Ba}}}{{\rm{Fe}}}} \right)_{{\rm{litho}}{\rm{.}}} - {\rm{p[P]}}_{{\rm{meas}}{\rm{.}}} \times \left(\frac{{\rm{Ba}}}{{\rm{P}}} \right)_{{\rm{bio}}{\rm{.}}}$$

where pBa_XS_, p[Ba]_meas._, p[Fe]_meas._, and p[P]_meas._ are measured in nM (nanomoles per liter); and Ba:Fe and Ba:P are molar abundance ratios of 2.87 mM:M and 0.44 mM:M, respectively (see Supplementary Note 1 for assignment of normalizing ratios). Particulate Ba_XS_ is thus the difference between supported (lithogenic- and organic matter-associated) and in situ pBa (Fig. 3a, e). Our partitioning indicates that pBa_XS_ constitutes 38% of the total upward-integrated pBa at WM and 45% at FWM, though these proportions increase to 45% (for WM) and 55% (for FWM) when considering only the depth ranges where organic matter attenuation exceeds −1 nM P m^−1^ (Fig. 3). Using this partitioning, we calculated the Ba-isotopic composition of pBa_XS_ from isotopic mass balance:

3 $${ {\delta}} ^{138/134}{\rm{pBa}}_{\rm{XS}} = ({ {\delta}} ^{138/134}{\rm{pBa}}_{{\rm{meas}}{\rm{.}}} - f_{{\rm{Ba}}_{{\rm{supp}}{\rm{.}}}} \times { {\delta}} ^{138/134}{\rm{pBa}}_{{\rm{supp}}{\rm{.}}})/f_{{\rm{Ba}}_{{\rm{XS}}}}$$

where *f*_*i*_ refers to the fraction of Ba in phase *i* (noting that $f_{{\rm{Ba}}_{{\rm{supp}}{\rm{.}}}} + f_{{\rm{Ba}}_{{\rm{XS}}}} = 1$); subscripts XS, meas., and supp., refer to pBa_XS_, total pBa (i.e., in situ), and supported pBa, respectively. We calculated δ^138/134^pBa_supp._ as ${ {\delta}} ^{138/134}{\rm{pBa}}_{{\rm{bio}}{\rm{.}}} \times f_{{\rm{Ba}}_{{\rm{bio}}{\rm{.}}}} + { {\delta}} ^{138/134}{\rm{pBa}}_{{\rm{litho}}{\rm{.}}} \times f_{{\rm{Ba}}_{{\rm{litho}}{\rm{.}}}}$, assuming that lithogenic matter was characterized by *δ*^138/134^Ba_NIST_ = +0.02 ± 0.05‰ (the mean ± 2 SD composition of particles closest to the lake bed; Fig. 2) and that organic matter-associated Ba has an isotopic composition of −0.02 ± 0.08‰ (mean ± range; see Supplementary Note 1). Uncertainties on p[Ba]_XS_ and δ^138/134^pBa_XS_ were calculated using a Monte Carlo method whereby each parameter in Eqs. 2 and 3 was (pseudo)randomly forced within its respective uncertainty and values of p[Ba]_XS_ and δ^138/134^pBa_XS_ calculated for each permutation. Final uncertainties reflect 2 SD of the probability distribution from 100,000 simulations. The mean Ba-isotopic composition of the pBa_XS_ for samples where $f_{{\rm{Ba}}_{{\rm{XS}}}} > 0.2$ was determined as −0.18 ± 0.09‰, equivalent to Δ^138/134^Ba_XS−diss._ = −0.41 ± 0.09‰ (±2 SE, *n* = 20; Fig. 3) relative to total dissolvable Ba in Lake Superior (=+0.23 ± 0.02‰; ±2 SD, *n* = 24).

### Evidence for pelagic barite precipitation in Lake Superior

The formation of an unsupported pBa excess, situated below peak p[P], bears striking similarity to modern marine pBa_XS_ distributions. However, modern marine barium cycling is paradoxical, in that p[Ba]^11^ and Ba-isotopic compositions^31^ are influenced by pelagic barite precipitation, yet seawater is almost everywhere undersaturated with respect to barite^15^. The most widely accepted model resolving this paradox posits that barite formation occurs within particle-associated microenvironments^11,12^ that develop during microbial oxidation of organic matter^38^. As oxidation proceeds, liberated Ba ions accumulate within microenvironments containing abundant seawater-derived sulfate until a critical level of supersaturation is achieved and barite precipitates^16,39^. Though these processes are extremely difficult to observe directly, five lines of evidence have been invoked to support this microenvironment-mediated model of pelagic barite formation in the marine water column: (a) close vertical association between barite formation and the depths of greatest organic matter attenuation;^12^ (b) positive correlation between the intensity of organic matter remineralization and the resultant pBa_XS_;^40,41^ (c) high and variable Ba:Sr ratios in pelagically precipitated barites^11^, reflecting variable contributions of organic matter-associated Ba and Sr to the microenvironment of barite formation; (d) observation of discrete μm-sized precipitates of barite in particulate matter;^11,12^ and (e) light Ba-isotopic compositions in precipitates relative to dissolved Ba in solution^30,31^. Critically, all five of these lines of evidence are satisfied by the particulate data from Lake Superior (Fig. 3), strongly supporting our hypothesis that pelagic barite precipitation is occurring despite micromolar ambient sulfate. We briefly explore each of these five lines of evidence before examining probable pathways to saturation when ambient conditions are undersaturated with respect to barite.

Firstly, we quantified the depth ranges of greatest organic matter attenuation by calculating the gradient of p[P] with respect to depth, which shows that the most intensive remineralization occurs between 12 and 30 m and 20 and 50 m at FWM and WM, respectively (Fig. 3b, f). That these exact depth ranges at their respective station also correspond to the largest increase in p[Ba]_XS_ would appear to support the vertical coupling between remineralization and the appearance of unsupported pBa. Secondly, the larger pBa_XS_ at FWM is consistent with both an increased intensity and quantity of organic matter remineralization compared with WM (c.f. *b*_FWM_ = −0.75 and *b*_WM_ = −0.55; Fig. 2a, e), in accord with observations of positive correlations between p[Ba]_XS_ and bacterial production in the open ocean^40^. Thirdly, p[Ba]:p[Sr] sharply increases with depth from approximately lithogenic values (~1.3) at the lake surface toward a maximum ≥2.5 M:M at the depths of peak p[Ba]_XS_ and greatest remineralization intensity, which is sustained into the hypolimnion (dissolved [Ba]:[Sr] is ≈0.34 M:M^42^). Fourthly, particulate samples collected from the DCM during cruise BH16-09 evidence mineral grains that are characteristic of barite in terms of size, habit, and X-ray emission spectra (Supplementary Figs. 4, 5, and 6; Supplementary Note 2). Though we did not obtain multi-element geochemical data for these filters, it is likely that these samples were collected at or close to peak p[Ba]_XS_ since the DCM in Lake Superior occurs well below the depths of peak primary production^22,29^. Fifthly, the Ba-isotopic compositions of the near-surface particles—and in particular those calculated for pBa_XS_—are isotopically lighter than total dissolvable (i.e., dissolved) Ba at both stations (Fig. 2d, h), with a similar average offset observed here (Δ^138/134^Ba_XS−diss._ = −0.41 ± 0.09‰) to that observed between dissolved Ba in seawater and barite-bearing particulates from the open ocean (Δ^138/134^Ba_part.−diss._≈−0.5‰; Supplementary Fig. 7). Thus, despite extreme barite undersaturation in Lake Superior waters, all five of the aforementioned lines of evidence used to invoke pelagic barite precipitation in seawater support our inference that similar processes are occuring in Lake Superior at dramatically lower ambient sulfate concentrations.

### Pathways to barite saturation

Achieving barite saturation in Lake Superior necessitates considerable deviation from equilibrium conditions, as ambient ${{\Omega }}_{{\rm{barite}}} \ll 1$. We contend that these deviations take place within protected microenvironments that form within aggregates of decaying organic matter. The ephemeral nature of particle-associated microenvironments makes direct observation extremely challenging, though their existence in open marine environments has been inferred from both biological^38^ and chemical^43^ techniques.

The formation of microenvironments first requires sufficient particle aggregation, which is itself a two-step process: particles must be brought into contact—either through purely physical (e.g., differential settling, Brownian motion, and shear) or biological (e.g., feeding) processes^44,45^—before they can be bonded together^46^. Once together, adhesion is dependent on both chemical and biological processes (e.g., products of cell lysis, extracellular polymers and exudates, and cell surface properties^46^). If sufficiently protected, microbial consumption and solubilization of organic matter within aggregates can eventually lead to the development of microenvironments that are chemically distinct from the surrounding solution^38^.

**Fig. 4:**
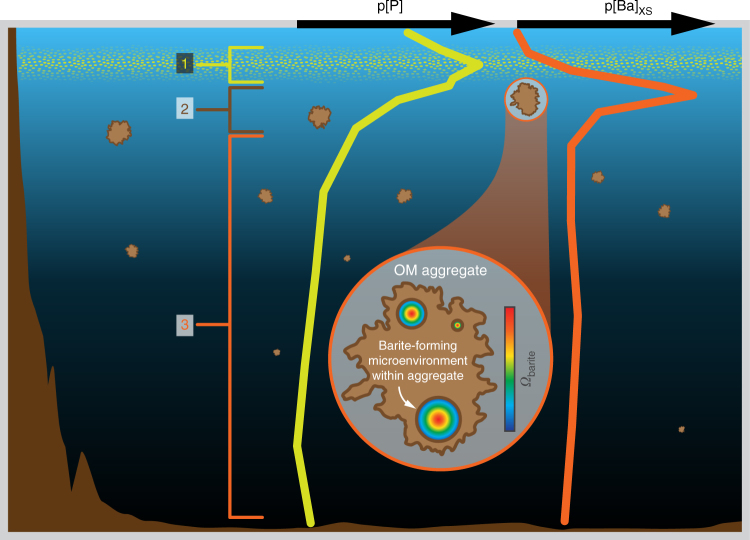
Conceptual model of barium cycling in low-sulfate water columns. Depth profiles of p[P] and p[Ba]_XS_ from St. FWM. (1) Autotrophic production leads to a peak in OM (organic matter) near the surface, indicated by the maximum in p[P]. (2) Microbial respiration within aggregates of decaying OM leads to development of barite-supersaturated microenvironments and precipitation of barite. (3) Continued respiration diminishes OM concentrations and destroys protected microenvironments, preventing further build up of Ba^2+^ and sulfate ions and thus any additional barite formation; settling barites are exposed to undersaturated waters and may start to dissolve

Crucially, Ba^2+^ and sulfate ions must be able to accumulate in these particle-associated microenvironments to such a degree that precipitation of barite becomes thermodynamically favorable (i.e., *Ω*_barite_ > 1; Fig. 4). Less crucial, however, is the type of organic matter supplying the Ba^2+^ and sulfate ions. Specifically, experiments conducted with non-axenic cultures demonstrated that barite formation occurred rapidly—regardless of the type of particle undergoing decay—and that diatoms, acantharia, and fecal pellets were not necessary precursors to barite precipitation^47^. These observations are important as they suggest that microenvironment-mediated pelagic barite precipitation is not dependent on the prevailing ecology but simply the presence of labile particulate organic matter and (micro)organisms capable of its degradation (e.g., ref. ^39^).

Continued respiration of organic matter will inevitably lead to microenvironment destruction and exposure of any barite precipitates to barite-undersaturated waters (Fig. 4). The extremely low solubility of barite, even in solutions where reduced sulfur species dominate total aqueous sulfur^6^, may enable some precipitates to settle out of the water column, either individually or within any remaining aggregates.

**Fig. 5:**
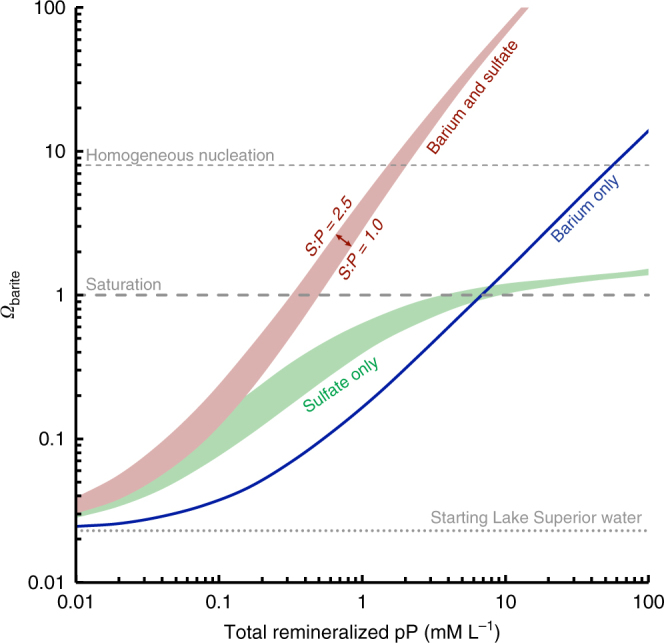
Barite saturation as a function of organic matter remineralization in Lake Superior. Curves illustrate the evolution of *Ω*_barite_ in 1-L solutions assuming that both Ba and sulfate, only sulfate, or only Ba are able to accumulate following remineralization. To achieve *Ω*_barite_ = 1 in the Ba and sulfate scenario, between 92 and 95% of sulfate and 67 and 75% of Ba ions in the environment of barite precipitation must be derived from remineralization. Though the absolute quantities of organic matter requiring remineralization are dependent on the geometry and aggregate volume of microenvironments, the proportionality determined by these calculations implicates respired organic matter as the dominant source of Ba and sulfate in driving pelagic barite saturation in Lake Superior

The development of a significant particulate Ba_XS_ coincident with the depths of strongest organic matter attenuation in Lake Superior is consistent with the remineralization of organic matter fueling pelagic barite precipitation (Fig. 3). To further explore this link, we performed a series of numerical simulations using PHREEQC^48^ whereby barium and sulfate ions were added to solution—together or individually—based on stoichiometric remineralization of organic matter (Fig. 5). The starting solution in each simulation was assumed to mimic the overall major ion chemistry of Lake Superior, initially with 40 μM sulfate^21^ and 69.7 nM Ba (this study), and with temperature (4.3 °C) and pH (8.11) taken from 18 m at FWM (corresponding to peak pBa_XS_); all modeling results are essentially identical for WM. Organic matter stoichiometry for Ba:P was fixed at 0.44 mM:M (Supplementary Note 1) and tested over a range of representative cyanobacterial S:P between 1.0 and 2.5 M:M^49^. These calculations were then used to place limits on the relative proportions of respired versus ambient Ba and sulfate in the microenvironments of barite formation. Calculation of the absolute quantities of mineralized organic matter necessary to achieve pelagic barite precipitation will require more sophisticated reaction—diffusion-type models that explicitly account for the chemical and physical characteristics of particulate microenvironments, which are beyond the scope of the present study.

Despite the simple approach used here, our simulations yield two significant results. Firstly, we find that barite saturation can be achieved with considerably less organic matter remineralization if both Ba and sulfate ions are able to accumulate in particulate microenvironments during respiration. If only one of either Ba or sulfate were able to accumulate, input of between 12 and 20 (if only sulfate) or 14 and 21 (if only Ba) times more respired organic matter would be required to achieve barite saturation compared to the Ba and sulfate scenario. Secondly, we calculate that to achieve saturation in a system where both ions are able to accumulate, between 67 and 75% of Ba and 92 and 95% of sulfate must be derived from respired organic matter, with the remainder sourced from ambient water (Fig. 5). Such high proportions of remineralized Ba and sulfate are conceptually consistent with the exceptionally low starting *Ω*_barite_ = 0.02 in Lake Superior, suggesting that the remineralization of organic matter and accumulation of respired ions in protected microenvironments is the critical process driving pelagic barite precipitation at micromolar ambient sulfate (Fig. 4).

### Barium-isotopic constraints on barite genesis

In the modern ocean, the flux of pelagic barite arriving at the seafloor is linked to the production and remineralization of organic matter in the water column^11,12,14^. Our observations in Lake Superior extend this linkage to ecosystems characterized by exceptionally low-sulfate concentrations (Fig. 4) and is therefore relevant to the debate surrounding the origin of ancient bedded barite deposits. For example, the formation of bedded barite in the Archean Aeon is considered enigmatic^5,20^ on account of the presumed approximately micromolar sulfate levels that characterized marine ecosystems at that time^17–19^. Since our observations in Lake Superior indicate that barites formed via pelagic precipitation at micromolar ambient sulfate possess Ba-isotopic compositions ≈−0.4‰ lighter than Ba in solution, it follows that ancient sedimentary barites should also exhibit Ba-isotopic compositions consistent with a heavy or seawater-like Ba source if formed via pelagic precipitation.

**Fig. 6:**
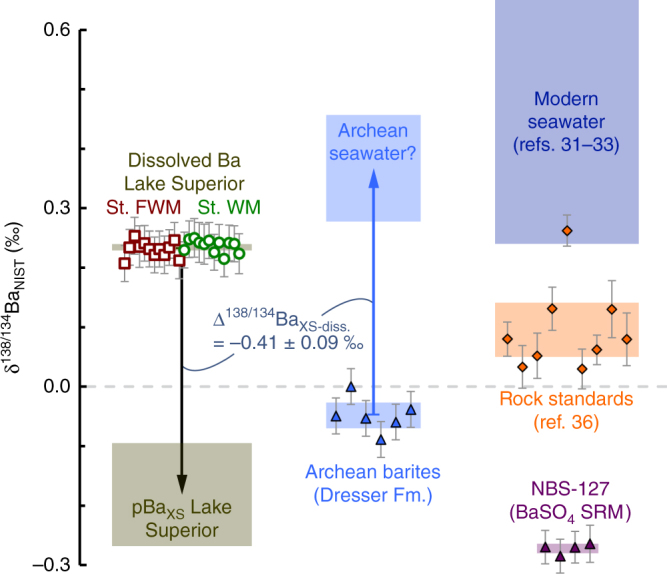
Isotopic offsets for barites precipitated under low ambient sulfate imply Archean seawater possessed heavy Ba-isotopic compositions. Using Δ^138/134^Ba_XS−diss._ = −0.41 ± 0.09‰ (this study), Archean barites from the Dresser Formation imply a dissolved Ba source with δ^138/134^Ba_NIST_ = +0.37 ± 0.09‰, similar to the range reported for modern seawater^31–33^, and considerably offset with respect to igneous rock standards (data are for AGV-1, G-2, BHVO-1, QLO-1, BIR-1, JG-1a, JB-1a, JR-1, and JA-1^36^). Values for NBS-127, a barite standard reference material commonly used for S- and O-isotopic normalization, were determined as δ^138/134^Ba_NIST_ = −0.27 ± 0.02‰ (±2 SD, *n* = 4), which we report here as a reference value for future studies. Shading indicates mean ± 2 SE for each sample set

We tested this model of barite formation by examining Ba-isotopic compositions of Paleoarchean barites from the Dresser Formation (Pilbara Craton, Western Australia; see “Sampling” section). This formation hosts the oldest bedded barites in the geological record (≈3.5 Ga^50^), though—as with other barite deposits from the Archean Aeon—their genesis is debated (e.g., refs. ^51,52^). Regardless, the geochemistry of bedded barites and attendant fluid inclusions in the Dresser Formation provides some of the earliest geochemical evidence for a thriving ecosystem, including sulfur-based microbial metabolisms^4,53^ and methanogenesis^54^. Our Ba-isotopic measurements of these barites yield δ^138/134^Ba_NIST_ = −0.05 ± 0.02‰ (±2 SE, *n* = 6), at least 0.1‰ lighter than lithogenic material (+0.10‰^36^; Fig. 6). This lighter-than-crustal Ba-isotopic composition for Dresser Formation barites appears to rule out their formation through hydrothermal baritization of original gypsum precipitates^4^ and allows for a pelagic influence in their precipitation as primary barite. Moreover, using our empirically determined offset of Δ^138/134^Ba_XS−diss._ = −0.41 ± 0.09‰ for pelagic barites in Lake Superior, we estimate that the Ba source to barites precipitated in the Dresser Formation possessed δ^138/134^Ba_NIST_ between 0.28 and 0.46‰, remarkably similar to the ranges observed in modern seawater (≈0.3–0.6‰^31–33^; Fig. 6). This inferred potential heavy Ba-isotopic composition of coeval seawater raises the tantalizing possibility that pelagic barite precipitation was a widespread process in the low-sulfate Paleorchean seas.

We further evaluated the feasibility of widespread pelagic barite precipitation in Earth’s early oceans, and its implications for primary productivity, by calculating order-of-magnitude Ba fluxes associated with pelagic barite precipitation at micromolar ambient sulfate from Lake Superior. Whole-lake estimates of primary production^22^ and its attendant respiration^55^ span 1–7 TM C per year, equivalent to an organic matter-associated Ba flux of 0.9–7.7 MM Ba per year if using our measured Ba:P stoichiometry of 0.44 mM and a representative C:P stoichiometry of 379:1 (for P-starved biomass^56^). Noting that between 25 and 33% of Ba in pelagic barite is sourced from dissolved Ba^2+^ (Fig. 5) and that ≈75% of barite precipitates are regenerated during export^57,58^, we estimate sedimentary fluxes in Lake Superior of 0.1–0.5 μg Ba cm^−2^ per year, somewhat lower than Ba fluxes measured below modern oligotrophic marine settings (0.5–1.0 μg Ba cm^−2^ year^59,60^).

Whether similar Ba flux estimates are reasonable for the low-sulfate Archean seas depends on several factors, such as the size of the contemporary biosphere and the flux of Ba entering the ocean at that time. If hydrothermalism were the dominant source of Ba to the oceans before the rise of continental weathering, and if Archean hydrothermal Ba fluxes were similar to (presumed) modern values, then removal of ≈3.4 GM Ba per year would be required to close the oceans’ Ba budget^58^. Using the proportionality between primary productivity and barite burial derived for Lake Superior, and assuming a more marine-like C:P of 106:1, our calculations suggest that a biosphere of ~2 PM C per year—approximately one-third of modern values (≈6.5 PM C per year^61^)—would be required to balance the Ba budget. Though this estimate is comparable with literature values (e.g., ref. ^62^), we urge caution in ascribing too much confidence to our estimate as it depends critically on the flux of Ba to seawater from hydrothermalism during the Archean, which could have been different to that (inferred for) today. Moreover, the concentration of dissolved Ba in Archean seawater was likely far higher than modern values^6^, which would suggest that our estimate of primary productivity is an upper limit. For example, elevated ambient [Ba] would favor higher organismal Ba:P^63^, contribute to a greater proportion of Ba in pelagic barite sourced from ambient dissolved Ba^2+^ (rather than organic matter), and increase barite preservation during export to the seafloor—all of these factors could lead to larger barite burial fluxes from a significantly smaller biosphere. Regardless, it is reassuring that our estimate is consistent with previous calculations, and suggests that pelagic barite precipitation in unproductive low-sulfate seas could have played an important role in the formation of enigmatic bedded barite deposits in the Archean Aeon.

## Discussion

Our finding that pelagic barite can precipitate at micromolar ambient sulfate has three important implications for interpreting the genesis, temporal distribution, and Ba-isotopic composition of barites in the sedimentary record. Firstly, low-ambient sulfate has hitherto been presumed such a significant thermodynamic barrier to barite nucleation that pelagic precipitates neither formed nor contributed to barite accumulations in ancient strata. However, our data strongly indicate that the thermodynamic barrier to barite precipitation presented by micromolar ambient sulfate is eminently surmountable if sufficient quantities of organic matter are remineralized in protected microenvironments. By analogy, we suggest that pelagic barites may too have precipitated in protected microenvironments in the water column when marine sulfate concentrations were in the micromolar range, such as during the Archaean^17–19^ or Neoproterozoic^64^. Extremely low barite solubility^6^ ensures that some proportion of pelagically formed precipitates will transit the water column and arrive at the seafloor, potentially contributing to sedimentary accumulations of enigmatic barites. More broadly, this model of barite precipitation highlights the importance of particulate microenvironments as microscopic mediators of chemical reactions that would otherwise be thermodynamically unfavorable at the macroscopic scale, raising the intriguing prospect that other cryptic biogeochemical transformations await discovery.

Secondly, our geochemical modeling reveals that in a system with only micromolar ambient sulfate, the vast majority of sulfate ions required to achieve barite saturation must be derived from respired organic matter in protected microenvironments. However, the emergence of microbes capable of sulfate reduction (after ~3.5 Ga^4^) would have dramatically altered the chemical landscape within these microenvironments, as any accumulated sulfate could henceforth be utilized as an oxidant to further mineralize organic matter. The net effect would be to reduce the amount of sulfate accumulating in particulate microenvironments, thereby depressing *Ω*_barite_ at the sites of pelagic barite formation and requiring significantly more organic matter remineralization to achieve barite saturation (akin to the Ba-only scenario; Fig. 5). It is thus tempting to speculate that the rise to ecological prominence of sulfate-reducing microbes ~3.0 Ga was the proximal cause of the ~1 Gyr hiatus in bedded barite sedimentation^5,20^ that only ended after the Great Oxidation Event (starting ≈ 2.44 Ga^65^), presumably as surface ocean concentrations of sulfate (and oxygen) rose to levels that enabled pelagic barite precipitation to resume.

Thirdly, Ba-isotopic analyses of sedimentary deposits may prove a powerful tool in discerning the environment under which enigmatic barites formed. Specifically, our results suggest that the Ba-isotopic composition of pelagic barites formed under low-sulfate conditions should exhibit Δ^138/134^Ba_XS−diss._≈−0.4‰ relative to dissolved Ba in solution. Even without estimates of contemporary dissolved δ^138/134^Ba_NIST_, such analyses may still provide valuable constraints on mineral genesis as the range of Ba-isotopic compositions encountered is likely directly proportional to the degree of environmental restriction during barite precipitation. Our measurements of deposits from the Dresser Formation—the oldest barites on Earth—appear to support a pelagic influence and are compositionally consistent with an open Ba reservoir with a heavy composition, such as seawater. These data provide the starting point for further investigations that utilize Ba-isotopic measurements of barites in the geological record, which are likely to yield new insights into mineral formation, seawater chemistry, and environmental conditions throughout Earth history.

## Methods

### Materials and methods

We followed recommended sampling and sample-handling protocols for GEOTRACES Cruises during sample collection aboard BH15-11, specifically those guidelines for collection of particulate samples from GO-Flo sampling bottles and for total dissolvable (i.e., unfiltered) samples (protocols available from http://www.geotraces.org/). These protocols were followed to the greatest extent possible before, during, and after BH15-11; thus, we only discuss the details of our sampling protocols when they necessarily deviated from guidelines.

The following plastics were used for sampling or during sample processing: HDPE (high-density polyethylene), LDPE (low-density polyethylene), PC (polycarbonate), PES (polyethersulfone), PFA (perfluoroalkoxy alkane), PLA (polylactic acid), PP (polypropylene), PTFE (polytetrafluoroethylene), and PU (polyurethane). All tubing, filters, sample containers, bottles, cubitainers, barbs, o-rings, connectors, and filter holders were acid-cleaned prior to use in a metal-free clean room at the NIRVANA Labs at WHOI (Woods Hole Oceanographic Institution); reagents used in cleaning solutions were typically of reagent-grade (or higher) quality, whereas reagents used for sample processing were strictly of environmental- or environmental-plus grade purity.

### Sampling

Sampling on Lake Superior was performed using the R/V *Blue Heron* during cruise BH15-11, which sailed from Duluth MN to Duluth MN during August 2015 (Fig. 1). Particulate sampling comprised one CTD (conductivitiy, temperature, and depth) cast at FWM and two casts at WM. At both stations, the initial downcast was used to select the 12 target depths in the water column at which samples would be collected; Nisken bottles were triggered at their respective target depths during the upcast only. The first water drawn from each Nisken was used to fill a 20 mL HDPE vial for analysis of total dissolvable Ba concentrations and Ba-isotopic compositions; the remaining ≈ 8 L of water was transferred to 12 × 10 L LDPE cubitainers. At FWM, the cubitainers were immediately taken below deck for filtration; at WM, this 8 L of water was instead used to rinse the interior of the cubitainers, then discarded. Following rinsing, a second CTD cast revisited the same 12 depths from the previous cast, with the 12 Nisken bottles triggered in the same order and at the same depths as before (±0.1 m). Sampled waters were then transferred to their respective cubitainer from the prior rinse cast, then taken below deck for filtering.

Hydrographic data from the CTD package are shown in Supplementary Fig. 1 for the particulate sample collection downcast only (first cast at FWM, event 002.02; second cast at WM, event 003.09); median downcast speed was 0.34 m s^−1^.

Samples were filtered immediately after collection using negative pressure from a central vacuum line. Each sample was drawn from a collapsible cubitainer through PU tubing, across a PES membrane filter (0.45 μm cutoff), and into a sealed container that would serve as the water gauge (≈10 L f-style HDPE container). Filters were held in place using PP Swinnex filter holders (EMD Millipore) and connected to the tubing lines with PC Luer taper fittings; filters were stored before and after use in sealed PC petri slide holders and manipulated using PP forceps. Each f-style water gauge container was connected to a vacuum manifold that ensured a negative pressure (and thus flow of water through the filter) and even distribution of negative pressure between all samples. Since the unfiltered samples from the CTD were drained from cubitainers, the containers would collapse as the samples were filtered, thereby maintaining a closed system and preventing nonnative particulates from entering the sampling line. Filtration proceeded for a minimum of 3 h or until the filters clogged, at which point the filters were removed from their holders, stored, and the water level in the f-style containers recorded; filtered volumes ranged from 0.5 to 3.0 L.

Three blanks were analyzed alongside the particulate filter samples: two process blanks and a dipped blank^26^. The process blanks were performed to assess the contributions from non particle-derived species that were acquired through sample processing, handling, and analysis. The dip-blank correction was used to account for species that were acquired through retention in the interstitial fluid of the membrane filter itself, or from adsorption of ions to the filter when in contact with lake water. The dip-blank contribution was assessed via clean dipping of an unused filter into Lake Superior using a purpose-designed PLA filter holder^66^ affixed to the CTD rosette during near-surface sampling at St. WM (PLA filter holder designed and fabricated by A.D. Thaler, Oceanography for Everyone; http://oceanographyforeveryone.com/); the dipped filter was subsequently stored for analysis and not used for active filtering.

Barite samples were collected from the Paleoarchean Dresser Formation at the North Pole Dome in northwestern Australia. Samples were taken from bedded barites exposed near the Dresser mine, as well as slightly down section from the stromatolite bearing cherts of the Buick locality. Only samples of bedded barite were collected; co-occurring vein barite that cuts through chert units in the area was avoided.

### Sample preparation prior to analysis

Particulate samples were prepared for analysis by leaching in 10 mL of 0.6 M HCl (hydrochloric acid) at 80 °C for ≥16 h. Treatment with 0.6 M HCl has a key benefit over more aggressive chemical treatments containing HNO_3_ (nitric) or HF (hydrofluoric) acids, in that leaching with 0.6 M HCl does not dissolve the membrane filter, which can contribute a significant proportion of the total Ba in a sample, particularly at low-ambient p[Ba]^67^. Furthermore, the fraction of Ba (also Ca, Cd, Mn, P, and Sr) recovered by leaching with 0.6 M HCl is equivalent to recovery using multi-acid total digestions^26^ but with considerably lower reagent-derived blank contributions.

Total dissolvable samples were weighed and subsequently acidified with HCl to pH ≈ 2) based on the mass of water within each HDPE container; samples and acid were left to equilibrate for ≥3 months before any further sample processing took place. The median proportion of Ba in the particulate phase as a fraction of the total was 0.13% (range 0.08–0.34%), in agreement with previous measurements^68^. Thus, total dissolvable [Ba] and Ba-isotopic compositions are equivalent to dissolved [Ba] and Ba-isotopic compositions within their respective analytical uncertainties.

Barite samples were mineralized using an alkaline dissolution in PTFE vials (after Breit et al.^69^). Approximately 10 mg of barite powder was drilled, homogenized, and weighed before dissolution. As per the original protocol, the amount of sodium carbonate added to each sample was adjusted to maintain a BaSO_4_:Na_2_CO_3_ mass ratio of 1:10; vials were topped up with 18.2 MΩ cm H_2_O to ensure that each sample contained 2 mL of solution per 10 mg of barite^69^. Following reagent addition, samples were sonicated for 60 min at room temperature before heating to 80 °C. After ≥16 h of reaction, samples were allowed to cool and the Na_2_SO_4_ liquid was decanted. This procedure was repeated three times in total. After the third decantation, samples were rinsed with 18.2 MΩ cm H_2_O and the remaining solid, BaCO_3_, was dissolved using 2 M HCl.

### Multi-element geochemical analyses

Sample leachates were analyzed for their multi-element geochemical compositions using a ThermoFisher ELEMENT 2 ICP-MS (inductively coupled plasma mass spectrometer) at the WHOI Plasma Facility. A 200 μL aliquot was subsampled from each of the 10 mL sample leachates, diluted to 1000 μL with 800 μL of 2% HNO_3_, and spiked with 100 μL of In (indium) solution—an internal standard added to all samples, standards, and blanks—to achieve a final [In] of 1 ng mL^−1^.

Elemental quantification in sample solutions was achieved via comparison of blank-corrected ion beam intensities to those of a reference curve constructed from measurement of eight standards with known concentrations (seven serially diluted standards plus origin). Ion beam intensities of Ba, Cd (cadmium), and Y (yttrium) were measured in low-mass resolution mode, whereas Al (aluminum), Ca (calcium), Fe, Mn (manganese), P, Sr, Ti (titanium), and V (vanadium) were measured in medium-mass resolution mode; In was monitored in both modes and internal normalization was performed separately for low- and medium-mass resolution elements. Analytical uncertainties from ICP-MS analysis refer to the propagated uncertainties from ion counting statistics, In normalization, and from the goodness-of-fit of the standard reference curve; typical uncertainties were between 2 and 4% for Al, Ba, Ca, Fe, Mn, P, Sr, and Y, and 7 and 10% for Cd, Ti, and V (±1 RSD; relative standard deviation).

Measured concentrations were converted to Lake Superior particulate concentrations by subtracting the blank from the leachate and then normalizing by the measured volume of water passed through each filter. Blank corrections were assumed to be independent of the volume filtered and were performed by subtracting the larger of the dipped blank (for Ba, Ca, Cd, Fe, Sr, Ti, and V) or the mean of the two process blanks (for Al, Mn, and P). The final uncertainty includes an additional, fully propagated term to account for the analytical uncertainties associated with measurement of the blanks themselves (median uncertainty ±12% RSD) but appropriately scaled to account for the minor magnitude of this correction (equivalent to a median correction of −3%).

### Barium-isotopic analyses

Before column chemistry, total dissolvable samples, particulate leachates, and barites were dried and spiked with an appropriate amount of ^135^Ba–^136^Ba double spike to ensure the ratio of spike to sample-derived Ba was between 1 and 2. Lake Superior samples were additionally fluxed in a 1:1 mixture of concentrated HNO_3_–H_2_O_2_ for ≥16 h at 135 °C to oxidize any organic matter in the sample that could interfere with Ba elution during column chemistry or during mass spectrometry itself. All samples were then converted back to Cl^−^ form and refluxed in 1 mL of 6 M HCl for ≥16 h to ensure complete spike–sample equilibration, at which point samples were twice passed through ion-exchange columns^31^ and subsequently redissolved in 2% HNO_3_ for Ba-isotopic analysis.

Barium-isotopic analyses were performed using a ThermoFisher Neptune multi-collector ICP-MS at the WHOI Plasma Facility. Samples were aspirated using a PFA micro-concentric nebulizer at rates ≈130 μL min^−1^, desolvated with a CETAC Aridus II, admixed with 3–5 mL min^−1^ N_2_, and introduced into the mass spectrometer operated in low-resolution mode, with Ba^+^ ion transmission efficiencies generally ≈1%. Our long-term uncertainty for sample unknown containing ≈50 ng of sample-derived Ba is typically ±0.03‰ (±2 SD^31^). Ascribing this level of uncertainty to the barite or total dissolvable water samples is warranted by the excellent agreement between the Ba-isotopic compositions of the 24 total dissolvable samples, which exhibit only ±0.02‰ variability (±2 SD) about the mean value of +0.23‰. However, the median quantity of sample-derived Ba in the particulate samples was generally less than half the amount present in total dissolvable samples (median 22 ng), such that our median analytical uncertainty for particulate samples was generally above ±0.03‰.

### Data availability

Hydrographic and geochemical data from Lake Superior are available through the Biological & Chemical Oceanography Data Management Office (deployment BH15-11; https://www.bco-dmo.org/deployment/685923;^70^). Barium-isotopic data for Dresser Formation and NBS-127 barites are tabulated in Supplementary Table 1.

## Electronic supplementary material

Supplementary Information
