# Publisher Correction: Pelagic barite precipitation at micromolar ambient sulfate

**DOI:** 10.1038/s41467-017-02701-y

**Published:** 2018-01-16

**Authors:** Tristan J. Horner, Helena V. Pryer, Sune G. Nielsen, Peter W. Crockford, Julia M. Gauglitz, Boswell A. Wing, Richard D. Ricketts

**Affiliations:** 1 NIRVANA Laboratories, Woods Hole Oceanographic Institution, Woods Hole, MA 02543-1050 USA; 2 Department of Marine Chemistry and Geochemistry, Woods Hole Oceanographic Institution, Woods Hole, MA 02543-1050 USA; 3 Department of Geology and Geophysics, Woods Hole Oceanographic Institution, Woods Hole, MA 02543-1050 USA; 4 Department of Earth and Planetary Sciences, McGill University, Montreal, QC Canada H3A 0E8; 5 Geological Sciences, University of Colorado Boulder, Boulder, CO 80309 USA; 6 Large Lakes Observatory, University of Minnesota Duluth, Duluth, MN 55812 USA

Correction to: *Nature Communications* 10.1038/s41467-017-01229-5, Article published online 07 November 2017

The original version of this Article contained an error in the barite saturation state equation in the fourth paragraph of the Introduction and incorrectly read ‘Ω_barite_=({^134^Ba^2+^}⋅{${\mathrm{SO}}_4^{2 - }$})/*K*_sp_)’. The correct version removes the superscript 134 next to ‘Ba^2+^’. This error has now been corrected in both the PDF and HTML versions of the Article.

