# Supplementary material for: Pelagic barite precipitation at micromolar ambient sulfate
Source: Nat Commun. 2017 Nov 7;8:1342. doi: 10.1038/s41467-017-01229-5 (PMC5673900; doi:10.1038/s41467-017-01229-5)
Supplement: Supplementary file 1 — Supplementary Information [file 41467_2017_1229_MOESM1_ESM.pdf]

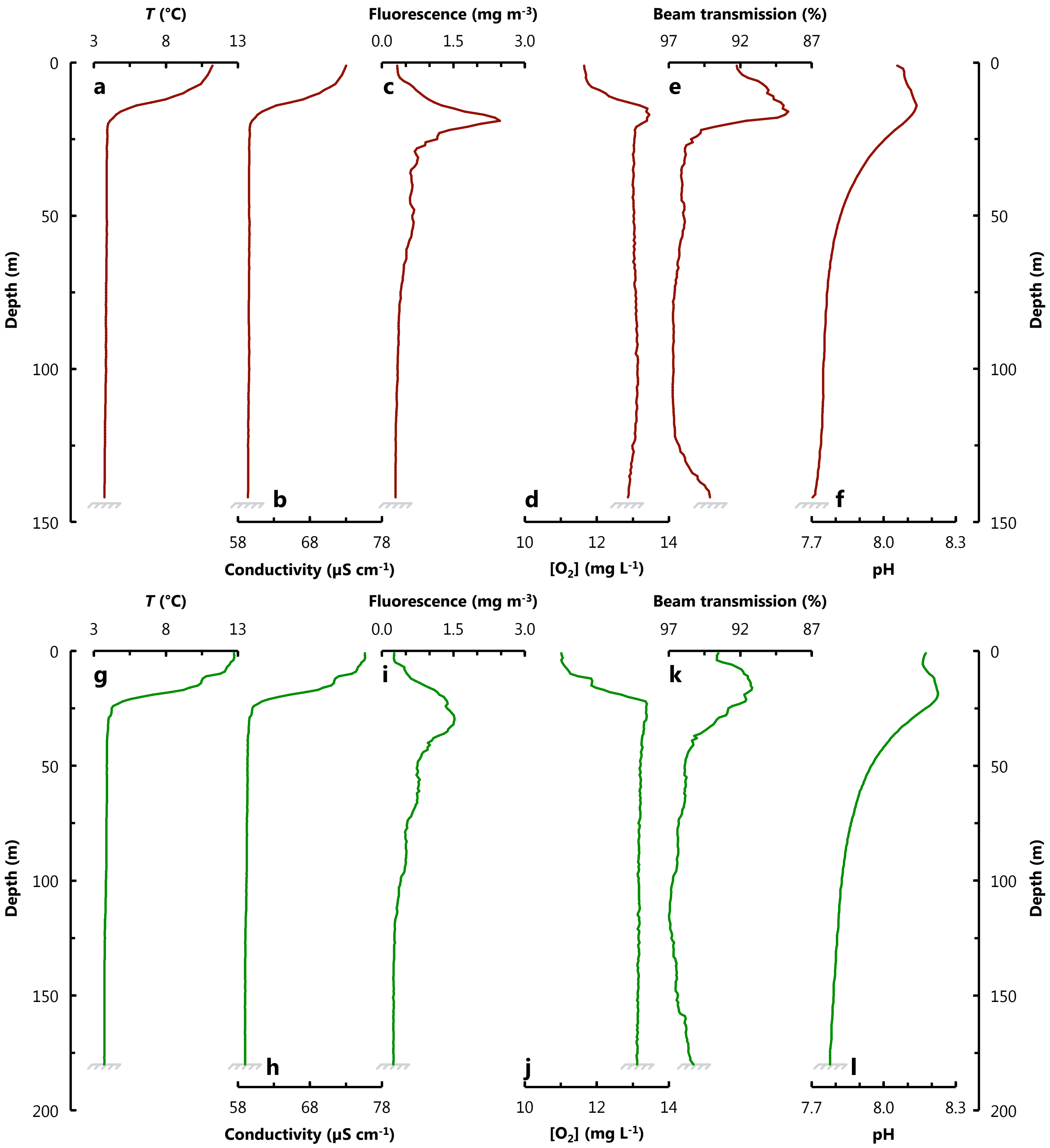

Supplementary Figure 1: Hydrographic data from BH15-11. Panels **a–f** from St. FWM; **g–l** from St. WM. Downcast profiles of **a, g** temperature; **b, h** conductivity; **c, i** fluorescence; **d, j** dissolved oxygen; **e, k** beam transmission (note reverse scale); and **f, l** pH. Data from individual scans have been binned into 1 m increments (median $= 70$ scans m^{-1}). Data are available from the Biological and Chemical Oceanography Data Management Office (deployment BH15-11).

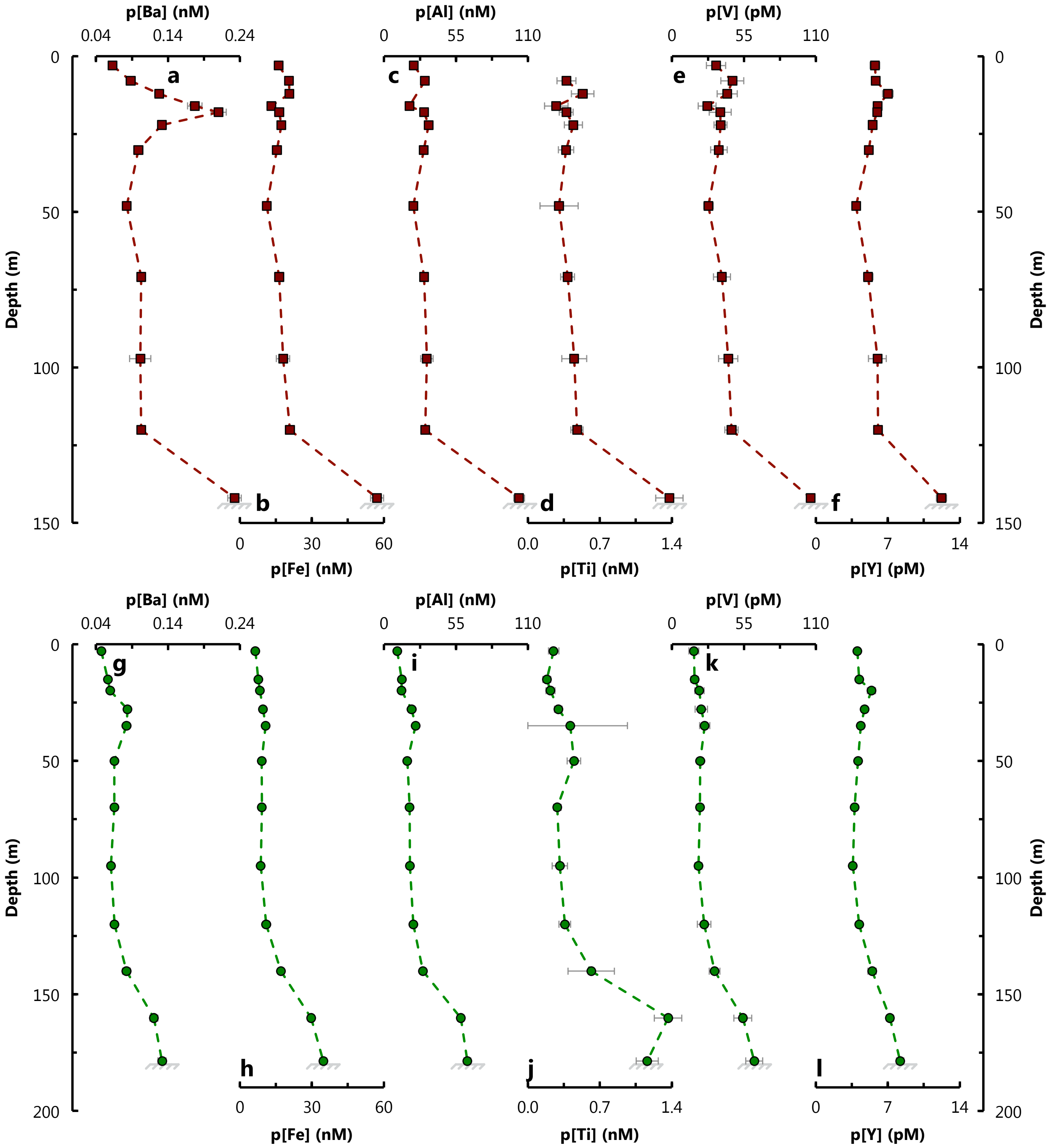

Supplementary Figure 2: Comparison of refractory/lithogenic tracer elements for Lake Superior. Panels **a–f** from St. FWM; **g–(l** from St. WM. Depth profiles of particulate **a, g** Ba; **b, h** Fe, our preferred tracer of lithogenic material; **c, i** Al; **d, j** Ti; **e, k** V; and **f, l** Y. Horizontal error bars for any given property measurement reflect the propagated 2× SD uncertainty. Data are available from the Biological & Chemical Oceanography Data Management Office.[1]

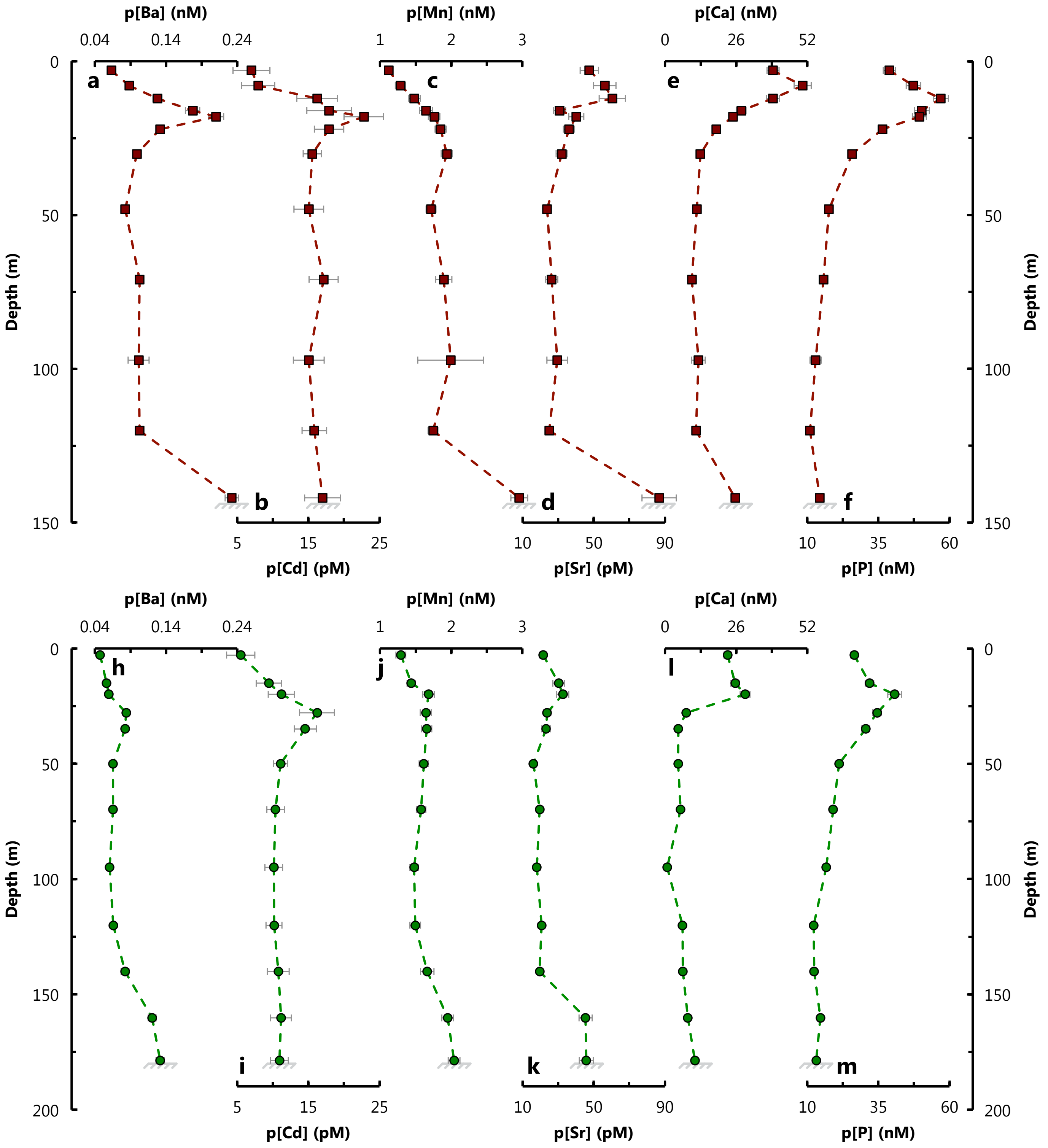

Supplementary Figure 3: Comparison of labile/biogenic tracer elements in Lake Superior. Panels **a–f** from St. FWM; **g–l** from St. WM. Depth profiles of particulate **a, g** Ba; **b, h** Cd; **c, i** Mn; **d, j** Sr; **e, k** Ca; and **f, l** P. Horizontal error bars for any given property measurement reflect the propagated 2× SD uncertainty. Data are available from the Biological & Chemical Oceanography Data Management Office.[1]

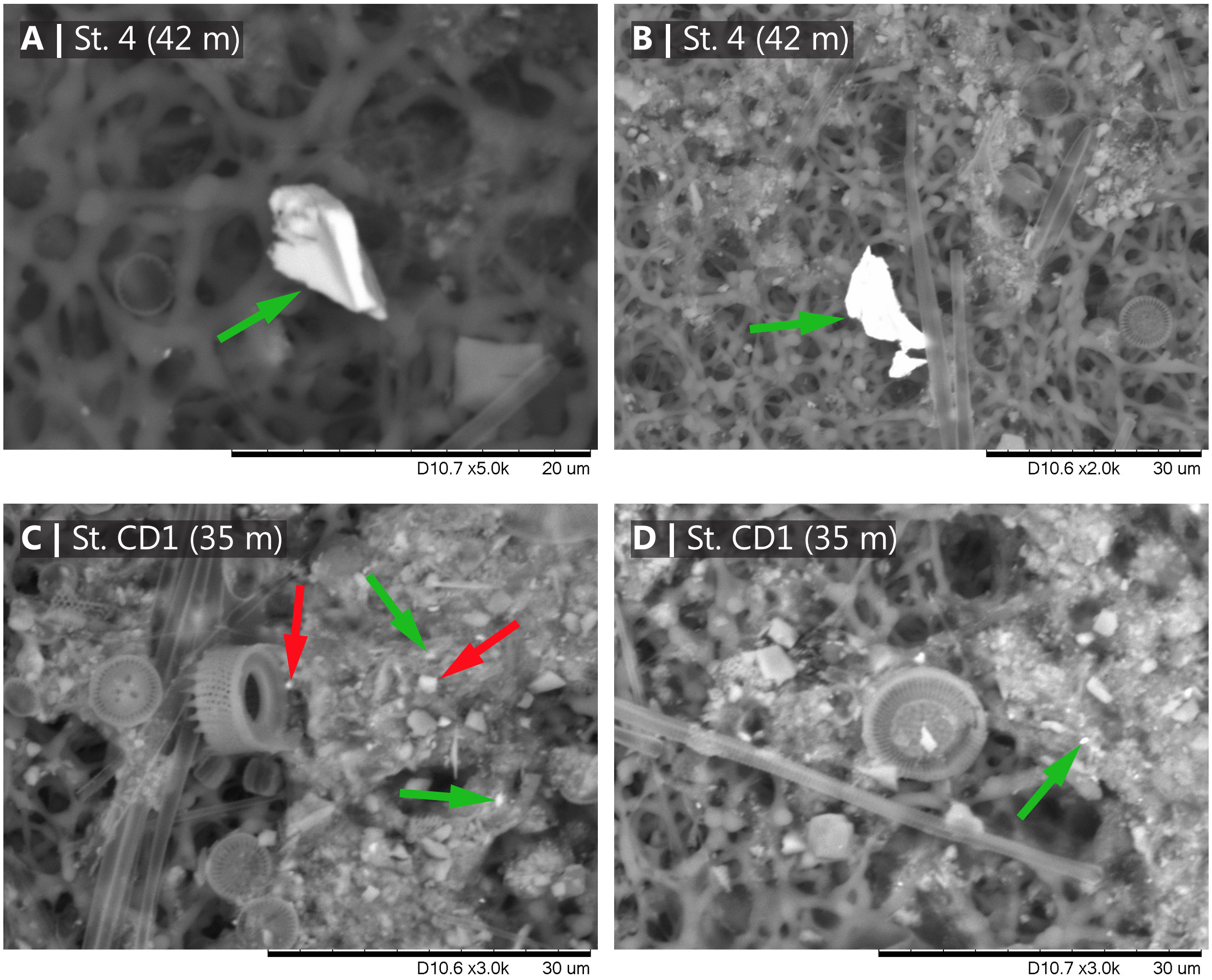

Supplementary Figure 4: Backscattered electron micrographs of filters collected during BH16-09. a, b ≈ 10 μm-size mineral grains from St. 4 and **c, d** $\approx 1\mu$m-size grains from St. CD1. Green and red arrows indicate putative barites and Fe-rich lithogenic particles, respectively, based on targeted elemental analyses (Supplementary Figs.5, 6). The brightness of the mineral grains relative to the groundmass (organic matter, plankton tests, and the filter itself) is indicative of the presence of heavy elements, such as Ba.

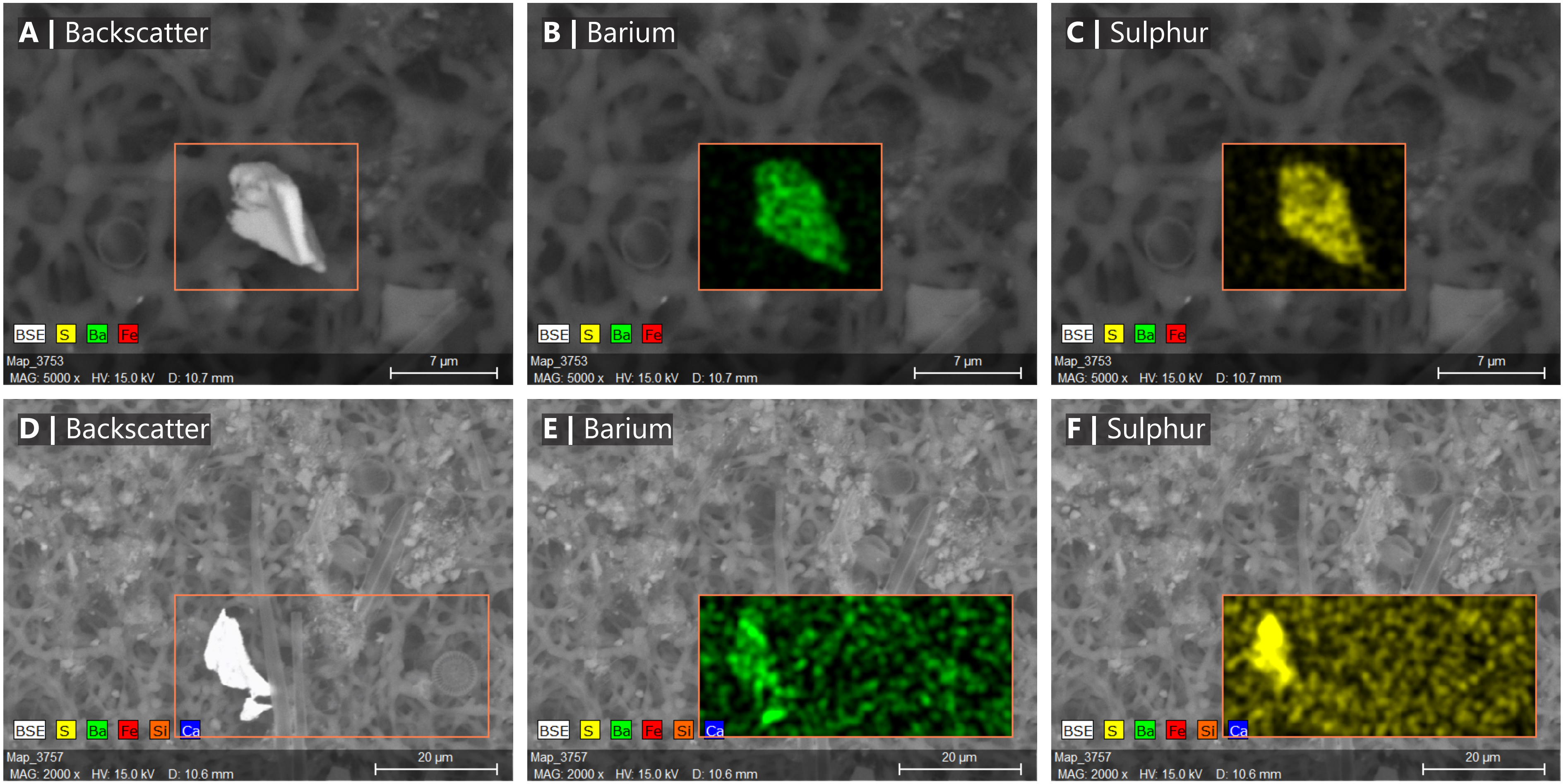

Supplementary Figure 5: Energy-dispersive X-ray spectrographic maps of Ba and S in grains from St. 4 (BH16-09). a, d Backscatter electron micrographs with box indicating areas mapped with EDS; **b, e** maps of Ba and **c, f** S. The co-location of Ba and S in these bright regions strongly suggests that the grains are barite ($BaSO_4$). Note that the topography of the filtered particulate matter and the oblique orientation of the EDS detector may lead to a slight mismatch in co-location between BSE micrographs and EDS maps.

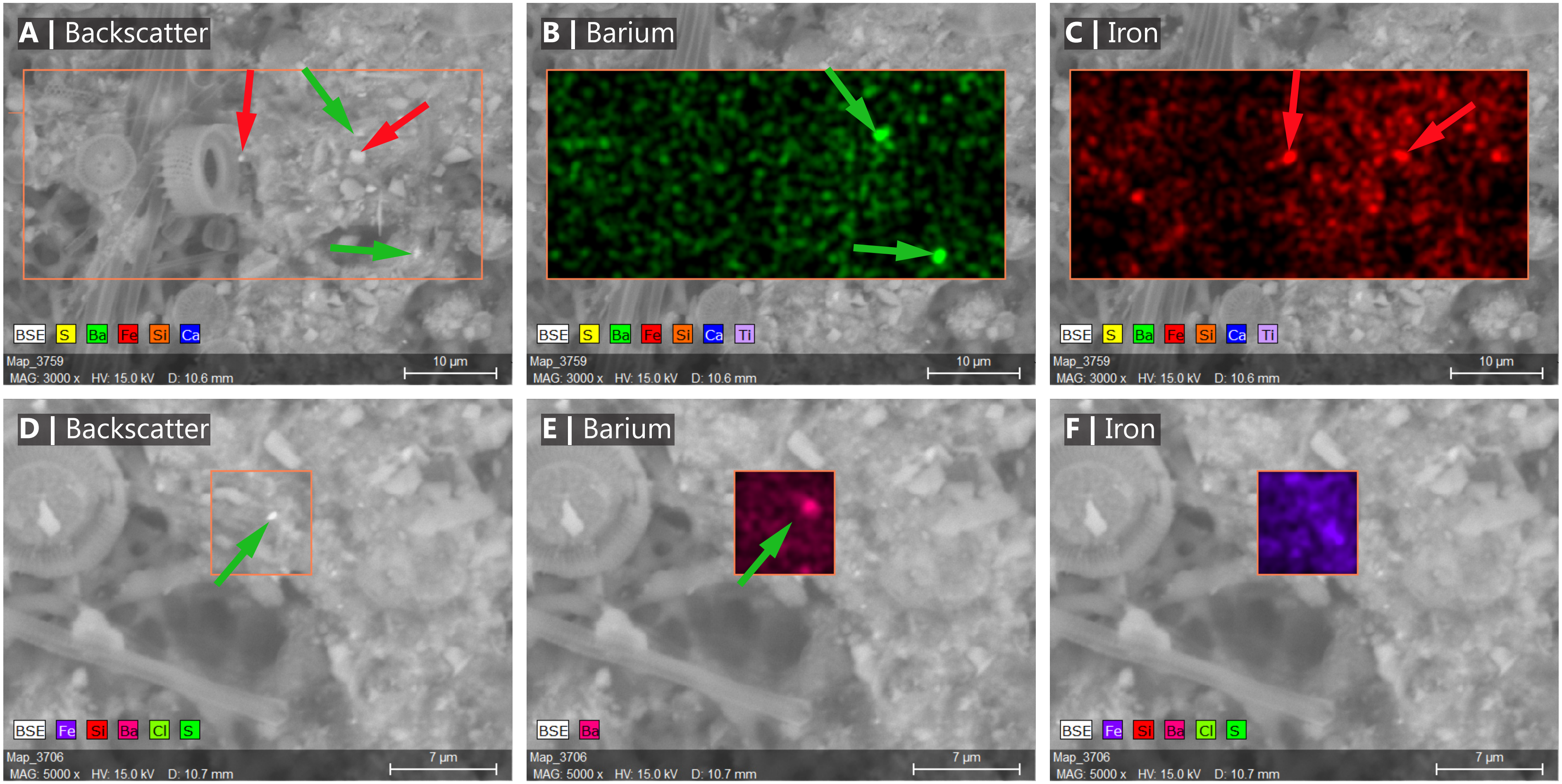

Supplementary Figure 6: Energy-dispersive X-ray spectrographic maps of Ba and Fe in grains from St. CD1 (BH16-09). a, d Backscatter electron micrographs with box indicating areas mapped with EDS; **b, e** maps of Ba and **c, f** Fe; arrows indicate grains identified under BSE (Supplementary Fig. 4). Sulphur could not be analyzed in these samples owing to the type of filter used. That Ba and Fe are not co-located supports the notion that the bright grains are not of terrigenous minerals lithogenic origin but rather barites. Note that the topography of the filtered particulate matter and the oblique orientation of the EDS detector may lead to a slight mismatch in co-location between BSE micrographs and EDS maps.

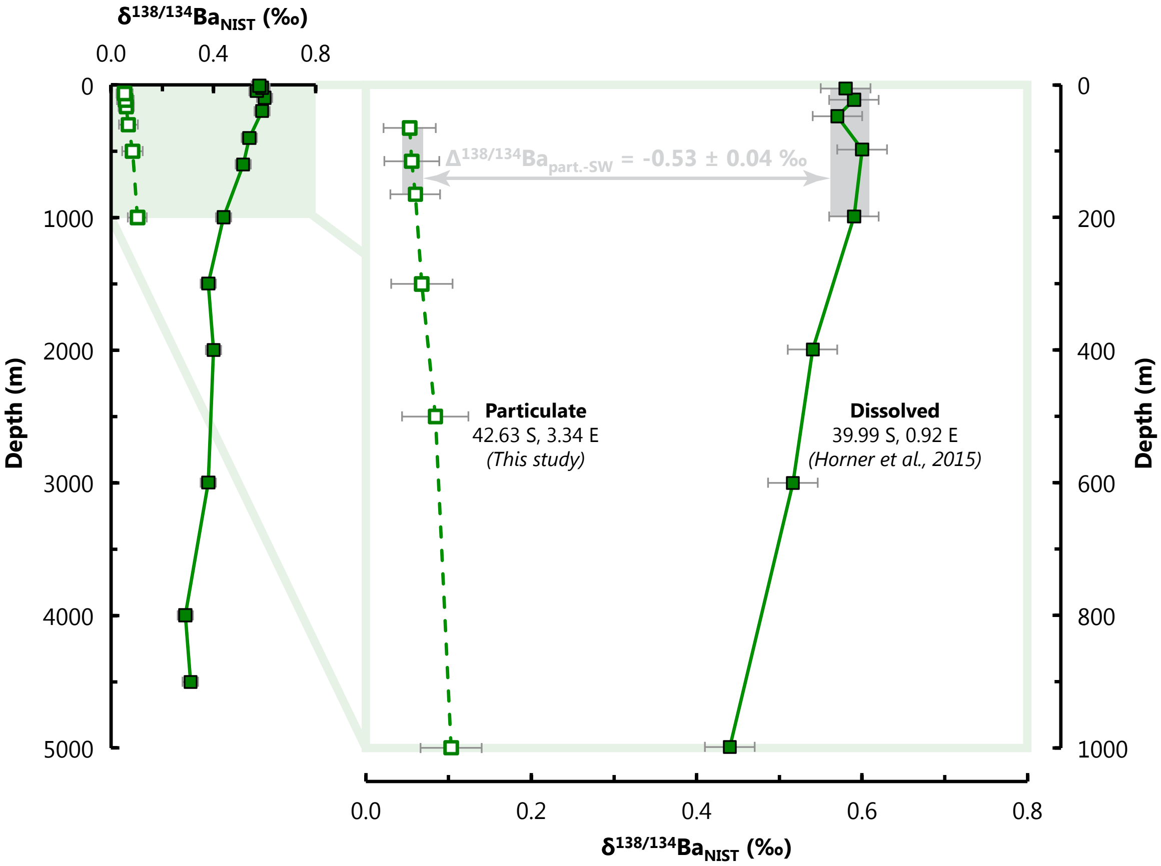

Supplementary Figure 7: Dissolved–particulate Ba-isotopic offsets from the South Atlantic. Particulate data are for $1 - 51$ μm size fraction collected via large volume *in situ* filtration at St. 109 of MV1101 (GCB1; *this study*); dissolved data from D357/GA10E.[2] Horizontal error bars for any given property measurement reflect the propagated $2\times$ SD uncertainty.

Supplementary Table 1: Barium-isotopic data for Archaean Barites and NBS-127 Reference Material.[3] See main text for dissolution procedure.

Sample Name	$\delta^{138/134}Ba_{NIST}$ (‰)	±2 SD (‰)
North Pole $BaSO_4$ # 1	−0.05	0.03
North Pole $BaSO_4$ # 2	0.00	0.03
North Pole $BaSO_4$ # 4	−0.05	0.03
North Pole $BaSO_4$ # 3	−0.09	0.03
North Pole $BaSO_4$ # 6	−0.06	0.03
North Pole $BaSO_4$ # 7	−0.04	0.03
Mean North Pole $BaSO_4$	−0.05 ± 0.02 ‰ (±2 SE)	
NBS-127 split a	−0.27	0.03
NBS-127 split b	−0.28	0.03
NBS-127 split c	−0.27	0.03
NBS-127 split d	−0.26	0.03
Mean NBS-127	−0.27 ± 0.01 ‰ (±2 SE)	

Supplementary Note 1

As noted in the main text, the particulate Ba excess, pBa_{XS}, was calculated as the difference between the 'supported' Ba (lithogenic- and organic matter-associated Ba) and measured (*in situ*) Ba concentration:

$$p[Ba]_{XS} = p[Ba]_{measured} - p[Fe]_{measured} \times \left(\frac{Ba}{Fe}\right)_{lithogenic} - p[P]_{measured} \times \left(\frac{Ba}{P}\right)_{biomass} \quad (1)$$

The following subsections deal with the considerations regarding the most robust proxy element for lithogenic material, then with the derivation of appropriate normalizing ratios for Ba : Fe and Ba : P (Eq. 1). In the discussion of ratios, we make two further assumptions that could affect the magnitude of pBa_{XS}. Firstly, we assume that the Ba contributions from lithogenic material (inferred using Ba : Fe) and organic matter-associated Ba (using Ba : P) cannot result in a negative pBa excess (i.e., $pBa_{XS} \geq 0$ nM), since excess-, lithogenic-, and organic matter-associated Ba must sum to *in situ* p[Ba]. In this formulation, positive values of pBa_{XS} do not therefore 'guarantee' the existence of barite, only whether, and in what quantity and unsupported pBa is present. Evidence for barite being responsible for the pBa_{XS} is instead derived from the depth range, relationship to organic matter remineralization, Ba : Sr, Ba-isotopic composition, and imagery of the particulates, which all provide strong evidence of pelagic barite precipitation.

Our second assumption relates to the choice of normalizing elements, Fe and P, as tracers of lithogenic material and organic matter, respectively. That is, we assume (i) values of p[Fe] are driven solely by the underlying distribution of lithogenic material and p[P] by organic matter, and (ii) the ratio of Ba : Fe in lithogenic material and Ba : P of organic matter is fixed in both space and time. Both of these assumptions likely oversimplify reality, as fixed ratios do not account for multiple particle types nor for processes that could alter Ba : Fe or Ba : P after a particle has formed, such as different lithogenic sources, chemical scavenging, or selective remineralization (e.g.,[4,5]). However, these assumptions appear to be of only secondary importance and serve only to alter the magnitude, but not the depth range or very existence of, the pBa_{XS} in Lake Superior.

Based on the full dataset of lithogenic-type tracers (Supplementary Fig. 2), potential caveats, and analytical considerations, we suggest that Fe is the best elemental proxy for lithogenic material in Lake Superior. Whilst it is possible that p[Fe] may be influenced by processes other than lithogenic cycling, such as biological assimilation or authigenic precipitation of Fe oxides, the excellent agreement between p[Fe] and other refractory elemental distributions indicates that p[Fe] primarily reflects the underlying distribution of terrigenous material in Lake Superior (Supplementary Figs. 1, 2). Indeed, basin-scale analysis of size-fractionated particulates collected from the North Atlantic indicates that the $1 - 51$ μm distributions of Fe is similar to several other elements, including Al, Th, Ti, V,

and Y, which are all overwhelmingly controlled by the abundance of lithogenic particles.[4] Moreover, examination of analytical uncertainties and potential blank contributions (from non lake-derived phases) all suggest that Fe is the most robust lithogenic tracer in Lake Superior. For example, our median analytical uncertainty was similar for Fe and Al (±2 %), increasing to ±3 % for Y, ±8 % for V, and ±10 % for Ti (±1 RSD). Further, the lowest median blank- : sample-derived elemental concentration ratios were obtained for Y ($\ll$ 1 %), then Fe (1 %), whereas Al, V, and Ti possessed values in excess of 3 %. Thus, the propagated uncertainty from analytical considerations and the background level of contamination indicates that Fe is the most robust overall tracer of lithogenic material in Lake Superior, though it is clear from the data in Supplementary Fig. 2 that normalization by any of Ba : Al, Ba : V, or Ba : Y in place of Ba : Fe in Eq. 2 would result in essentially identical distributions of pBa_{XS}.

It has been reported that leaching with 0.6 M HCl may underestimate total particulate concentrations for elements hosted in refractory phases, such as lithogenic matter, compared with multi-acid attack. However, this effect is relatively minor for samples above 255 m depth,[6] well below the deepest depths sampled here of $\leq$ 180 m (St. WM). As discussed above, the excellent agreement between elements that are predominantly hosted in refractory phases (Supplementary Fig. 2) essentially precludes any element specific undersampling bias, as we would expect this bias to manifest as divergent depth profiles for the elements hosted in refractory phases, with the degree of divergence controlled by the relative solubility of the host phase(s) in 0.6 M HCl. The absence of this effect, the shallow depth ranges sampled, and the highly-systematic nature of the profiles indicates that elemental recovery is near-quantitative for all Lake Superior particulate samples.

The Ba : Fe used here to calculate lithogenic-associated Ba was 2.87 mM : M, which is slightly lower than the range of average continental crust Ba : Fe of $4.0 - 8.8$ mM : M (ref.[7]). That the Ba : Fe of particulates in Lake Superior is slightly lower than the crustal average is not surprising since the watershed surrounding Lake Superior contains numerous banded iron formations (e.g.,[8,9]), which possess significant proportions of Fe and thus may contribute to lower-than-average Ba : Fe ratios in fluvially-derived particles entering Lake Superior. The lowest observed Ba : Fe ratio during our sampling was 3.79 ± 0.22 mM : M (±2 SD), measured within the core of the benthic nepheloid layer $\approx$ 180 m at St. WM (Supplementary Fig. 1). Though this benthic bottom layer is predominantly composed of resuspended terrigenous material (e.g.,[10]), 13.1 nM of pP were also measured in this sample (Supplementary Fig. 3), which likely contribute some organic matter-associated Ba to the *in situ* total pBa. Thus, the 'true' Ba : Fe ratio of lithogenic material must be $<$ 3.79 mM : M to satisfy the reqiurement that $pBa_{XS} \geq 0$; use of Ba : Fe = 2.87 mM : M satisfies this requirement for all samples when using Ba : P = 0.43 mM : M, discussed next.

The Ba : P used here for organic matter-associated Ba was 0.44 mM : M, which is of a similar magnitude to near-surface marine particles collected during the GEOTRACES NAZT ($\approx$ 0.6 mM : M; North Atlantic Zonal Transect[4])

and for cultured plankton (≈ 0.4 mM : M;[11]). However, it is worth noting caveats specific to each of these estimates of Ba : P. Firstly, *in situ* bulk particles likely overestimate organismal Ba : P as these samples invariably contain lithogenic material and/or barite, which both contribute significant quantities of Ba (but generally not P;[2]). Indeed, there does not appear to be any indication of a significant biologically-associated component to particulate Ba cycling along the NAZT.[4] Secondly, more recent culturing experiments employing chelating agents to buffer free metal concentrations to marine-relevant levels yielded significantly lower organic-associated Ba : P of of 0.5 μM : M.[12] Thus, it highly likely that true organismal Ba : P is $\ll 1$ mM : M. In the absence of culture data for freshwater organisms we assigned organismal Ba : P based on our own observations from Lake Superior. Using analogous reasoning as for assigning Ba : Fe of lithogenic material, the 'true' value for Ba : P must be less than or equal to the lowest observed value.

The lowest particulate Ba : P observed here was 0.44 mM : M., which was obtained for a zooplankton sample collected at St. WM by winching a net from 100 m depth to the surface at ~ 10 m min^{-1}, filtering $\approx 78,500$ L of water. (A similar net tow was performed at St. FWM that yielded Ba : P $= 0.76$ mM : M.) The 64 μm mesh of the zooplankton net was utilized for estimating the Ba : P and Ba-isotopic composition of organic matter as typically both barite and lithogenic particles occur in the $1 - 51$ μm size range.[4,13] Thus, the zooplankton net tow may circumvent some of the Ba contributions from non organic matter-associated phases and, in the absence of culture data, provides the most reasonable estimate of organismal Ba : P and $\delta^{138/134}$Ba$_{NIST}$ for Lake Superior. As noted above, the 'true' value for organismal Ba : P must be less than or equal to the lowest observed value to account for any extraneous lithogenic or barite particles; we thus use the value obtained for St. WM of 0.44 mM : M in our calculation of p[Ba]$_{XS}$.

For the calculation of $\delta^{138/134}$pBa$_{XS}$, we use the mean Ba-isotopic composition of the two zooplankton net tows of $\delta^{138/134}$Ba$_{NIST} = -0.02$ ‰ (values for St. WM and FWM were determined separately as $\delta^{138/134}$Ba$_{NIST} = -0.10 \pm 0.03$ ‰ and $+0.06 \pm 0.03$ ‰, respectively). In estimating the uncertainty on $\delta^{138/134}$pBa$_{XS}$, we allowed the mean organic matter-associated Ba-isotopic composition of -0.02 to vary by the total range observed in Lake Superior (± 0.08 ‰). Though this represents a large uncertainty on the Ba-isotopic composition of organic-matter associated Ba, the overall insignificance of this phase to the total particulate Ba budget renders a comparatively smaller uncertainty in $\delta^{138/134}$pBa$_{XS}$.

Supplementary Note 2

Here we present representative BSE (backscatter electron) micrographs and EDS (energy-dispersive X-ray spectrography) maps of filters that evidence the presence of barite in Lake Superior particulate matter. Filters from BH15-11 were necessarily dissolved for the purposes of obtaining multi-element geochemical and Ba-isotopic data. Thus, the filters imaged in Supplementary Figs. 4, 5, and 6 were collected during a subsequent expedition that sailed in June 2016 (BH16-09; Milwaukee WI–Duluth MN).

Particulate collection during BH16-09 targeted the DCM, as this feature was found to correspond to the highest pBa_{XS} at St. WM and FWM during BH15-11. Sampling took place at two stations: St. 4 (46°55′13.7″ N, 86°35′55.1″ W; 2016-06-28) and within the vicinity of St. CD1 (47°03′44.7″ N, 91°24′27.4″ W; 2016-06-30). Reconnaissance CTD casts were used at both stations to identify the location of the DCM–42 m and 35 m at St. 4 and CD1, respectively–before repositioning the ship to the original coordinates and deploying large volume *in situ* pumps. All filter holders were prepped in the shipboard laboratory space and covered with polyethylene bags until immediately before deployment. A series of tiered 142 mm filters were used to collect particles in different size classes (from highest to lowest): 150 μm nylon, 51 μm PES (polyethersulphone), 3 μm PES or acrylic copolymer, and 0.22 μm PES. An aspirator pump was used to remove water from the filter head before filters were removed using PTFE forceps and stored frozen until analysis.

Electron microscopy was performed at Woods Hole using a Hitachi TM-3000 Tabletop Microscope operated with a 15 kV acceleration voltage; representative BSE micrographs from St. 4 and CD1 are shown in Supplementary Fig. 4. Candidate barite grains were subsequently analyzed by EDS using a Bruker Quantax 70 Energy-Dispersive X-ray Spectrometer; representative maps from St. 4 and CD1 are shown in Supplementary Figs. 5 and 6, respectively. The characteristic x-ray emissions lines for Ba ($L\alpha \approx 4.47$ keV) and Ti ($K\alpha \approx 4.51$ keV) are sufficiently similar that the two elements are difficult to resolve using 'tabletop' EDS. Distinguishing between Ba and Ti is an important consideration for establishing the presence of barite in the samples. This issue can be overcome by comparing Ba elemental distributions in mineral grains with those of other elements likely to be co-located with Ba in barite, such as S (sulphur).

At St. 4 it is clear that Ba and S are co-located in the bright grains identified using BSE, strongly indicative of the presence of barite (Supplementary Fig. 5). At St. CD1 it was not possible to analyze S as the membrane used to filter particles from the lake was composed of PES, necessitating use of an alternative indicator element. Our profile data from BH15-11 indicate that p[Fe] and p[Ti] distributions are dominated by the presence of lithogenic matter (Supplementary Fig. 2). It thus follows that Fe and Ti should co-occur in certain grains of lithogenic origin, whereas

authigenic barites should exhibit Ba but not Fe (or Ti). Though this test is less assured than if measuring S directly–and relies on Fe and Ti co-occurring in the same minerals–we nonetheless identified numerous grains that were either Ba (or Ti)-rich or Fe-rich at CD1 (Supplementary Fig. 6). When considered in the context of the μm-size range and 'bright' characteristics of the grains when imaged using BSE, these additional tests are entirely consistent with the presence of barite in these samples.

Lastly, we note that these images are of secondary significance to the other geochemical data that evidences barite precipitation, since the identification of barite in Lake Superior particulate matter does not address the manner of precipitation. That is, the distinction between *in situ* (i.e., pelagically precipitated) versus *ex situ* barites (e.g., eroded from the surrounding watershed and carried into the lake via fluvial processes) cannot be made by BSE micrographs or EDS maps: the geochemical tests prescribed in the main text are the primary means by which the origin of the pBa_{XS} can be discerned.

Supplementary References

[1] Horner, T. J., Ricketts, R. D. & Pryer, H. V. Particulate multi-element geochemical concentrations, dissolved barium concentrations and barium-isotopic data collected during the R/V *Blue Heron* cruise BH15-11 in Lake Superior during August 2015 (2017). doi:10.1575/1912/bco-dmo.680972.

[2] Horner, T. J., Kinsley, C. W. & Nielsen, S. G. Barium-isotopic fractionation in seawater mediated by barite cycling and oceanic circulation. *Earth Planet. Sci. Lett.* **430**, 511–522 (2015).

[3] Halas, S. & Szaran, J. Improved thermal decomposition of sulfates to SO_2 and mass spectrometric determination of δ^{34}S of IAEA SO-5, IAEA SO-6 and NBS-127 sulfate standards. *Rapid Commun. Mass Spectrom.* **15**, 1618–1620 (2001).

[4] Ohnemus, D. C. & Lam, P. J. Cycling of lithogenic marine particles in the US GEOTRACES North Atlantic transect. *Deep Sea Res. Part II* **116**, 283–302 (2015).

[5] Twining, B. S. *et al.* Differential remineralization of major and trace elements in sinking diatoms. *Limnol. Oceanogr.* **59**, 689–704 (2014).

[6] Planquette, H. & Sherrell, R. M. Sampling for particulate trace element determination using water sampling bottles: methodology and comparison to *in situ* pumps. *Limnol. Oceanogr. Methods* **10**, 367–388 (2012).

[7] Wedepohl, K. H. The composition of the continental crust. *Geochim. Cosmochim Acta* **59**, 1217–1232 (1995).

[8] Huston, D. L. & Logan, G. A. Barite, BIFs and bugs: evidence for the evolution of the Earth's early hydrosphere. *Earth Planet. Sci. Lett.* **220**, 41–55 (2004).

[9] Rasmussen, B. *et al.* Deposition of 1.88-billion-year-old iron formations as a consequence of rapid crustal growth. *Nature* **484**, 498–501 (2012).

[10] Halfman, B. M. & Johnson, T. C. Surface and benthic nepheloid layers in the western arm of Lake Superior, 1983. *J. Great Lakes Res.* **15**, 15–25 (1989).

[11] Fisher, N. S., Guillard, R. R. & Bankston, D. C. The accumulation of barium by marine phytoplankton grown in culture. *J. Mar. Res.* **49**, 339–354 (1991).

[12] Sternberg, E., Tang, D., Ho, T. Y., Jeandel, C. & Morel, F. M. M. Barium uptake and adsorption in diatoms. *Geochim. Cosmochim. Acta* **69**, 2745–2752 (2005).

[13] Dehairs, F., Chesselet, R. & Jedwab, J. Discrete suspended particles of barite and the barium cycle in the open ocean. *Earth Planet. Sci. Lett.* **49**, 528–550 (1980).
